# Coagulopathy following major trauma hemorrhage: lytic, lethal and a lack of fibrinogen

**DOI:** 10.1186/cc13923

**Published:** 2014-06-16

**Authors:** Ross Davenport

**Affiliations:** 1 Centre for Trauma Sciences, Blizard Institute, Bart’s and the London School of Medicine & Dentistry, Queen Mary University of London, 4 Newark Street, London E1 2AT, UK

## Abstract

Trauma-induced coagulopathy (TIC) is present soon after injury and is associated with increased transfusion requirements and worse outcomes. The pathophysiological mechanisms, which result in the widespread derangements of hemostasis following major trauma hemorrhage, are as yet not fully defined. Profound activation of fibrinolytic pathways and fibrinogen depletion appear to be fundamental processes in the development of TIC and offer potential therapeutic targets. Collaborative and multi-disciplinary scientific study is thus a research priority in order to characterize the primary drivers of TIC and develop targeted and efficacious treatment strategies.

## 

Trauma-induced coagulopathy (TIC) exacerbates bleeding after severe injury and is associated with poor outcomes. In the previous issue of *Critical Care*, Oshiro and colleagues [1] showed that changes in hemostatic competency occur early following trauma and are described by a profound derangement in coagulation, in particular massive activation of fibrinolysis and potentially fibrinogenolysis. In the context of the CRASH-2 (Clinical Randomization of an Antifibrinolytic in Significant Hemorrhage 2) trial [2], which showed a mortality benefit with the use of the anti-fibrinolytic tranexamic acid (TXA) in trauma hemorrhage, this work further highlights the pathophysiological importance of fibrinolysis in TIC. Early detection of clotting deficiencies appears to have prognostic value, and the authors provide evidence for the utility of a scoring system in predicting outcome and transfusion need [1]. However, a definitive mechanism for TIC and thus optimal therapeutic interventions are currently unknown. Investigation of pathophysiological pathways and identification of efficacious, targeted treatment strategies therefore remain a research priority.

Mechanistic understanding of acute hypocoagulability and early hypercoagulability following major trauma in recent years has moved away from the simplistic and reductionist theories of the lethal triad (hypothermia, dilution, and acidosis). The cell-based model of hemostasis, an intimate relationship between coagulation and inflammation, platelet function, fibrinogen availability, and importance of the endothelium have shaped current hypotheses implicating tissue hypoperfusion and tissue trauma as potential drivers for TIC [3]. In a large prospective study of trauma patients, with serial blood sampling, Oshiro and colleagues have reaffirmed the presence of an early coagulopathy of trauma and have shown it to be defined predominantly by a fibrinolytic phenotype with increased fibrinogen breakdown [1]. Through application of the Japanese Association for Acute Medicine disseminated intravascular coagulation scoring system obtained immediately after arrival, the authors have demonstrated a close association with an alternative definition of post-traumatic clotting derangement: acute coagulopathy of trauma shock. In addition, the scoring system used has been shown to be an independent predictor of prognosis and the need for massive transfusion.

Unfortunately, historical definitions and difficulties in reaching consensus on nomenclature to describe early coagulation changes in trauma have prohibited any quantum leaps in our understanding of the disease process. Scoring systems in trauma and coagulation are numerous yet function only as surrogates for disease-specific markers. Global collaboration and coordinated study by hematologists, experts in transfusion medicine, trauma surgeons, and critical care physicians are required for a comprehensive scientific investigation of TIC. Both European and US collaborations appear to have answered this call to arms with two large-scale research initiatives both entitled TACTIC: the European Commission-funded Targeted Action for Curing TIC [4] and the US Trans-Agency Research Consortium for TIC [5]. The objectives of both collaborations are to support multi-component scientific research to conduct hypothesis-driven studies of TIC, link clinical investigators with basic scientists, and fund trauma-related clinical trials of TIC.

Recent years have seen a handful of large international clinical trials of TXA (CRASH-2) [2], recombinant Factor VIIa [6], and fresh frozen plasma (FFP)/platelets (Pragmatic Randomized Optimal Platelet and Plasma Ratios, or PROPPR) [7] founded on a limited mechanistic understanding of TIC. However, among researchers there is a growing consensus, supported by the study by Oshiro and colleagues [1], that both fibrinogen availability and inhibition of fibrinolysis are essential components for hemostasis. Evidence from the trauma community, supported by basic science and research in other disciplines in which major hemorrhage is a frequent occurrence (that is, cardiac surgery and obstetrics), has reached broad agreement of the necessity to maintain fibrinogen at more than 1.5 g/L [8]. Studies have shown it falls fastest and most significantly during trauma hemorrhage [9,10] compared with other clotting factors. More importantly, fibrinogen supplementation with either cryoprecipitate (UK or US) or fibrinogen concentrate (Europe) is often delayed or considered second-line in the empiric delivery of hemostatic coagulation therapy and therefore replacement fails to keep up with depletion. Consequently, early- and high-dose fibrinogen replacement is the subject of two pilot randomized controlled trials in the UK (Early Cryoprecipitate for Severe Trauma Haemorrhage Trial, or CRYOSTAT) [11] and Austria (Fibrinogen Concentrate in Trauma Patients, Presumed to Bleed, or FiTIC) [12] due to report later this year.

In agreement with Oshiro and colleagues [1], previous observational studies have shown a high prevalence of fibrinolytic activation after major trauma [13,14], but doubt remains as to the best method of rapid detection. Currently, viscoelastic hemostasis assays - for example, thromboelastography or rotational thromboelastometry - are the only point-of-care measures which can detect fibrinolysis, although both are relatively insensitive measures of clot breakdown. However, CRASH-2 [2] and the large body of evidence from both elective and emergency surgery have shown TXA to be a safe and efficacious treatment for hemorrhage without assay-proven evidence of fibrinolysis. The mechanism of fibrinogen depletion is unclear, and fibrinogen cleavage by plasmin is difficult to measure, although hypothetically direct fibrinogenolysis may explain the rapid and significant loss of fibrinogen during massive activation of fibrinolytic pathways.

In this study, Oshiro and colleagues [1] have demonstrated the importance of fibrinolysis and potentially fibrinogenolysis in the pathogenesis of early traumatic coagulopathy. However, further work is required to fully delineate early coagulopathy following major trauma to achieve a better understanding of the mechanistic drivers and develop targeted treatment strategies. Empiric transfusion algorithms do not improve TIC during active hemorrhage [15], and the efficacy of FFP is unclear. Early treatment with TXA is associated with mortality benefit in trauma hemorrhage, and early fibrinogen replacement could potentially augment correction of TIC, reduce transfusion requirements, and improve outcomes. The challenge remains to determine how best to stratify those at risk of major bleeding and which patients are most likely to benefit from early treatment. Coordinated, cross-disciplinary efforts at unraveling the complexities of the coagulation system are required to best identify therapeutic targets and improve outcomes from major trauma hemorrhage.

## Abbreviations

CRASH-2: Clinical Randomisation of an Antifibrinolytic in Significant Hemorrhage 2; FFP: fresh frozen plasma; TIC: trauma-induced coagulopathy; TXA: tranexamic acid.

## Competing interests

The author declares that he has no competing interests.
